# Risk of SARS-CoV-2 Infection in Previously Infected and Non-Infected Cohorts of Health Workers at High Risk of Exposure

**DOI:** 10.3390/jcm10091968

**Published:** 2021-05-04

**Authors:** Adrián Sánchez-Montalvá, Candela Fernández-Naval, Andrés Antón, Xavier Durà, Alba Vimes, Aroa Silgado, Fernando Velásquez-Orozco, Juan Espinosa-Pereiro, Fernando Salvador, Tomás Pumarola, Benito Almirante, Juliana Esperalba

**Affiliations:** 1 Infectious Diseases Department, Vall d’Hebron University Hospital, International Health Program of the Catalan Insitute of Health (PROSICS), Universitat Autònoma de Barcelona, 08035 Barcelona, Spain; xavierduramiralles@gmail.com (X.D.); jespinosa@vhebron.net (J.E.-P.); fmsalvad@vhebron.net (F.S.); balmiran@vhebron.net (B.A.); 2 Microbiology Department, Vall d’Hebron University Hospital, Vall d’Hebron Barcelona Hospital Campus, Universitat Autònoma de Barcelona, 08035 Barcelona, Spain; candela.fernandez@vhir.org (C.F.-N.); aanton@vhebron.net (A.A.); aroa.silgado@vhir.org (A.S.); fvelasquez@vhebron.net (F.V.-O.); tpumarola@vhebron.net (T.P.); jesperalba@vhebron.net (J.E.); 3 Clinical Pharmacology Department, Vall d’Hebron University Hospital, Vall d’Hebron Barcelona Hospital Campus, Universitat Autònoma de Barcelona, 08193 Barcelona, Spain; avimes@vhebron.net

**Keywords:** reinfection, SARS-CoV-2, natural immunity, genome amplification diagnostic assays, serology

## Abstract

The objective of this study is to assess the risk of newly acquired RNA detection-proven SARS-CoV-2 infection after previous SARS-CoV-2 infection. This is a prospective study conducted from March to September 2020 in Barcelona, Spain. Healthcare workers caring for SARS-CoV-2 infected patients were divided in two cohorts: (a) previously RNA-proven SARS-CoV-2 infected cohort with mild symptoms (IC) and (b) healthy cohort (HC). Weekly SARS-CoV-2 RNA detection assays from nasopharyngeal swabs were performed. Serology status was assessed at the beginning and at the end of the study. Twenty participants were included in each group. The median age was 30 (IQR 27–34.75) years, and 55% were female. The median time of follow up was 49 (IQR 49–51) days. Fifteen out of 246 (6%) nasopharyngeal swab samples were positive for SARS-CoV-2, all in the IC. The percentage of participants in the IC with a probable newly acquired SARS-CoV-2 RNA-proven infection was 20% (95% IC 5.7–43.6%) at the end of the 7-week follow up period. The incidence reinfection rate was 28.6 (95% IC 7.8–73.2) cases per 1000 person-week. Despite detectable IgG antibodies against SARS-CoV-2 participants highly exposed to SARS-CoV-2 may develop a newly acquired SARS-CoV-2 RNA detection episode during the first months after the initial infection.

## 1. Introduction

The new coronavirus, SARS-CoV-2, is having devastating consequences in health systems, global economy, and social dynamics and behavior. Despite the huge efforts invested to fight the pandemic, SARS-CoV-2 still runs wild with the current death toll as high as the first months of the pandemic [1]. Non-pharmacological strategies are effective to curb the pandemic, although cannot be held too long without causing a negative impact in the economy and social health [2,3].

It is well described that herd effect can control highly infective diseases. However, the protection generated by the immune system needs to fulfil several criteria in order to adequately protect the society. Firstly, it should be a long-standing immunity. Secondly, it should have the capacity to reduce transmissibility (reducing the basic reproduction number) by limiting the contagious power or hindering the acquisition of the infection. Thirdly, antigens stimulating the immune system should be highly conserved hampering the appearance of new strains able to escape the immune response [4].

Immunity against SARS-CoV-2 can be naturally acquired or vaccine-induced. There is still scarce evidence on whether the immunity against SARS-CoV-2 reduces disease severity, infection and/or transmissibility. Equally important is the duration of the immunity, as short duration of protection will jeopardize proper control of the disease. To date, time from infection to decay of anti-SARS-CoV-2 antibodies and the role of these anti-bodies to prevent a new infection in naturally infected subjects are a matter of intense debate. Based on the current evidence it seems probable that naturally acquired immunity against SARS-CoV-2 confers early strong protection that may wane over time [5,6,7]. No data is available on whether vaccines can develop a longer immune response and protection than natural infection [8].

Long standing immunity after exposure to SARS-CoV-2 antigens, naturally or artificially via vaccine, with capacity to reduce disease severity and transmissibility remains the cornerstone of the strategy to fight the pandemic. For that reason, performing a thoroughly investigation of newly acquired infections after previous SARS-CoV-2 infection is of utmost importance. Reinfection cases have been anecdotally reported all over the world ranging from asymptomatic to severe cases requiring hospitalization and oxygen supplementation [9]. Unfortunately, it has been scarcely evaluated in a cohort of participants highly exposed to the virus [10]. Healthcare workers in Spain were badly hit by the pandemic during the first wave with infection proportions ranging from 25.8% to 33.8% in a cross-sectional study performed in May 2020 [11]. Healthcare workers highly exposed to the SARS-CoV-2 represent an outstanding opportunity to study risk of reinfection.

In our study we aimed to assess assessed the risk of newly acquired RNA detection-proven SARS-CoV-2 infection in two highly exposed cohorts of healthcare workers, one with a previous history of RNA detection-proven SARS-CoV-2 infection and another with no previous history of RNA detection-proven SARS-CoV-2 infection during a period of 7 weeks. Secondary objective was to assess the severity of a newly acquired RNA detection-proven SARS-CoV-2 infection and the correlation with IgG antibodies levels.

## 2. Materials and Methods

### 2.1. Study Setting and Population

This is a prospective cohort study conducted from March 2020 to September 2020 at Vall d’Hebron University Hospital, Barcelona, Spain. Participants were health care workers accompanying SARS-CoV-2 infected patients. According to the previous history of SARS-CoV-2 infection, participants were divided in two cohorts: (a) previously infected cohort (IC), which included subjects with viral symptoms 7 days prior to detection of SARS-CoV-2 RNA from a nasopharyngeal swab and (b) healthy cohort (HC), which included subjects without history of viral symptoms and no record of previous RNA detection-proven SARS-CoV-2 infection or antibodies against the SARS-CoV-2. Subjects from IC cohort were invited to participate during the first two weeks after returning to work from the isolation period.

### 2.2. Study Procedures

After signing the informed consent, all participants fulfilled a structured questionnaire to collect demographic data, previous medical history and medication, viral symptoms in the previous weeks and work conditions and locations. Participants were instructed how to report symptoms during the study period in an individual’s symptoms diary. All participants were followed for 7 weeks. Additional consent was requested to gather self-reported data after the end of the study.

According to the study protocol, a blood sample for serology assessment was collected at baseline and at the end of the 7-week follow up. A nasopharyngeal swab was collected at baseline and every week until the end of the study. Nasopharyngeal swab collection could be either performed by a member of the research team or self-collected by the participant after training. Samples were received at the laboratory within the first 4 h after sample collection for their immediate storage in a −80 °C freezer. Blood samples were kept at −20 °C before the serological testing. An extra nasopharyngeal swab collection was performed if the patient reported viral symptoms during the study period.

In the IC cohort, a probable newly acquired SARS-CoV-2 infection was suspected if a participant had a positive SARS-CoV-2 RNA detection assay at least 30 days after symptoms onset and had at least one negative SARS-CoV-2 RNA detection assay between the initial and the new positive assay.

### 2.3. Statistical Analysis and Sample Size Calculation

Continuous variables were expressed as median and interquartile range and categorical variables as absolute numbers and percentages. Longitudinal results are depicted in a timeline graphic for clarity. PCR-proven SARS-CoV-2 infection incidence comparison among groups was performed using proportion difference test. Tests were considered significant when the two-tailed *p*-value was <0.05. For the sample size calculations, we assumed a proportion of new PCR-proven SARS-CoV-2 infection after the 7-week period of 25% and 0% in the HC and the IC group, respectively. Given an alpha error of 0.1, a beta error of 0.2, and an exposure ratio of 1 the sample size was 21 participants per arm. Analysis was performed with SPSS software (IBM^®^, Armonk, New York, USA).

### 2.4. Study Oversight and Ethical Statement

The institutional review board provided ethical clearance (PR (AG) (195/2020)). All patients signed a written informed consent.

### 2.5. Microbiology Procedures

#### 2.5.1. SARS-CoV-2 RNA detection

The detection of SARS-CoV-2 in nasopharyngeal samples was performed using the commercial Aptima^®^ SARS-CoV-2 Assay (Hologic, Marlborough, Ma, USA), which utilizes transcription-mediated amplification (TMA) for nucleic acid amplification on the Panther Fusion^®^ System (Hologic, USA). This amplification technique is known to have a higher sensitivity than conventional PCR-based assays in other RNA-viral infections [12]. TMA was the first-line assay due to the high throughput of the system. As the results obtained by TMA-based techniques do not have correspondence with viral quantification load, and additional real time PCR-based assay was performed for the determination of the cycle-threshold (Ct). Moreover, this technique was also used to assess the sample collection, integrity of extracted nucleic acids and presence of PCR inhibitors by detecting of a human housekeeping gene. Thus, the positive samples by TMA-based assay were additionally tested using an in-house RT-PCR assay based on the CDC 2019-nCoV Real-Time-PCR Diagnostic Panel, with two sets of primers and probe targeting the specific SARS-CoV-2 nucleocapsid protein (N1) gene and the human RNase P gene.

#### 2.5.2. Determination of Humoral Immune Response against SARS-CoV-2 

Serological response to SARS-CoV-2 was determined by the detection of IgG antibodies against spike SARS-CoV-2 spike glycoprotein using the commercial enzyme-linked immunosorbent assay (ELISA) anti-SARS-CoV-2 ELISA (IgG) (EUROIMMUN, Lübeck, Germany) performed on the EUROIMMUN Analyzer I-2P (EUROIMMUN, Lübeck, Germany). 

## 3. Results

### 3.1. Population Description

Twenty participants were included in each group. The median age of the participants was 30 (IQR 27–34.75) years, and 55% were female. One participant in the IC was under anti-TNF treatment due to a joint inflammatory disease and one participant in the IC became pregnant at the end of the study but adhered to the protocol schedule. In the IC cohort, the median time from the first PCR-proven SARS-CoV-2 episode to inclusion in the study was 22.5 (IQ 18–25) days. All participants in the IC cohort reported mild symptoms, and none of them required hospitalization. During the follow up, all participants continued working at COVID19 high-risk hospital areas. The median time of follow up was 49 days (IQR 49–51) with five participants lost to follow up. No participants in either group reported new symptoms during the 7-week study period. Seven participants spontaneously reported extra data beyond the study time. More information can be found in Table 1.

### 3.2. SARS-CoV-2 RNA Detection

Overall, 15 out of 246 (6%) nasopharyngeal swab samples were positive by TMA. All the 123 follow up samples from the HC were negative. In the IC, 15 out of 123 (12%) follow up samples were positive (including the post study follow up self-reporting period). Seven out of 20 (35%) participants in the IC had at least one positive result during the follow up. Seven participants (35%) had a positive SARS-CoV-2 RNA detection beyond 30 days after inclusion in the study. At the time of the inclusion in the study, 4 participants had a positive SARS-CoV-2 RNA detection, which was considered as remnant from the first PCR-proven infection.

**Figure 1:**
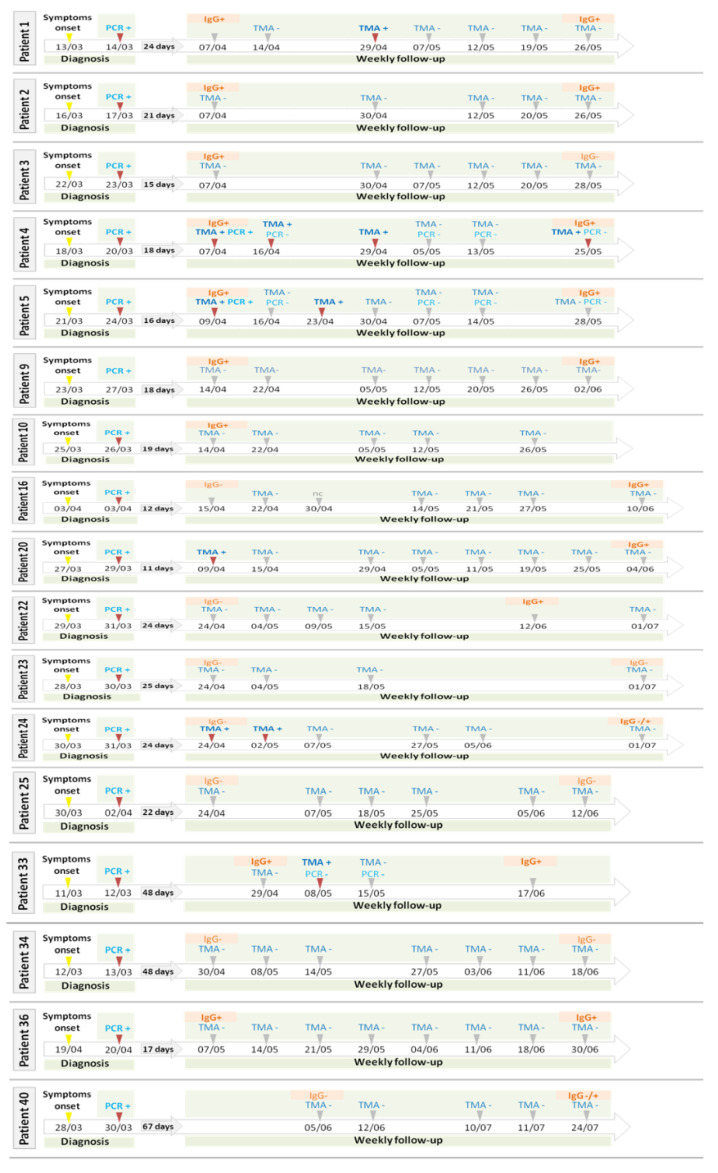
Timeline of participants with no evidence of newly acquired infection or early probable reinfection in the IC cohort. TMA: transcription-mediated amplification, PCR: polymerase chain reaction.

**Figure 2:**
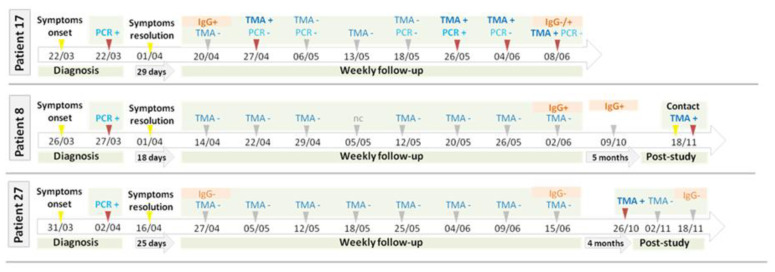
Timeline of participants with late newly acquired infection. TMA: transcription-mediated amplification, PCR: polymerase chain reaction.

According to our case definition, the percentage of participants in the IC with a probable newly acquired SARS-CoV-2 RNA-proven infection was 20% (95%IC 5.7–43.6%) at the end of the 7-week follow up period, with 2 additional cases in the self-reporting period, which depicted 30% (95% IC 11.9–54.3%) of probable newly acquired SARS-CoV-2 RNA-proven infection in 8 months. The incidence reinfection rate, taking into consideration the 7-week study period, was 28.6 (95% IC 7.8–73.2) cases per 1000 person-week in health care workers at high risk of SARS-CoV-2 exposure. None of them had symptoms at the time of RNA detection of the second probable infection, and no subsequent infections in close contact were declared. Figure 1 and Figure 2 depict the timeline for symptoms and testing results of participants in the IC. Additional information regarding these cases is available in Appendix A. When comparing the percentage of newly acquired SARS-CoV-2 RNA detection proven infection between HC and IC we did not find any statistically different (0% vs. 20%; *p =* 0.11).

### 3.3. Serology Results

At the time of inclusion, 18 out of 20 participants in the IC had an available initial serology, and 55.6% (10/18) had specific IgG antibodies against SARS-CoV-2. At the end of the 7-week study 11 out of 17 (64.7%) had positive IgG antibodies against SARS-CoV-2. Four participants had negative IgG antibodies against SARS-CoV-2 throughout the duration of the study, 4 participants seroconverted, and 1 participant had undetectable IgG antibodies against SARS-CoV-2 at the end of the study period.

All participants in the HC had negative IgG antibodies against SARS-CoV-2 at the beginning and at the end of the study, except for one participant who had a positive anti-SARS-CoV-2 IgG at the end of the study, although all 8 RT-PCR from nasopharyngeal swabs were negative during the study period. This participant had positive total antibodies against SARS-CoV-2 at the time of inclusion with negative IgG antibodies, emphasizing the possibility of a recent asymptomatic SARS-CoV-2 infection before participating in the study.

## 4. Discussion

In this report, we describe a prospective study involving 40 health care workers taking care of COVID19 patients. Twenty participants had been previously diagnosed with symptomatic SARS-CoV-2 infection confirmed by molecular tests and 20 participants without evidence of previous SARS-CoV-2 infection were included as controls. In the HC group, all participants had RNA-detection assays and IgG antibodies against SARS-CoV-2 negative at the beginning of the study. Based on our definition of probable newly acquired SARS-CoV-2 infection, 4 participants could be classified probable newly acquired SARS-CoV-2 infection during the 7-week study period; therefore, the incidence rate of reinfection in the 7-week study was 28.6 cases 1000 person-week in our study population. When including the self-reporting period, 6 participants fulfilled the probable newly acquired SARS-CoV-2 infection.

Persistence of detectable of SARS-CoV-2 RNA in nasopharyngeal samples has been reported for long periods, especially in patients with impaired immune systems. However, follow up periods are usually short, covering only the first weeks of the infection [13,14]. In a recent report by Kim et al., hospitalized participants with COVID19 were repeatedly sampled to assess viral shedding and viability in viral culture. From symptoms onset, the median time to SARS-CoV-2 RT-PCR clearance occurred on day 34 (lower limit of 95% CI being the 24th day, upper limit was not computable) in 50% of the patients [15]. In our study the 4 participants with probable newly SARS-CoV-2 infections during the 7-week study had at least 55 days between the onset of symptoms and the SARS-CoV-2 RNA detection assay that led to reinfection suspicion, and 3 out of these 4 probable cases of reinfection had several negative RNA tests in between, reinforcing the likelihood of reinfections rather than remnants from the first infection. The cases 8, 17 and 27 deserve special attention since they had TMA-positive swab nasopharyngeal samples several months after the first episode. In patient 17, RT-PCR was also positive with Ct values consistent with an acute infection. Patient 8 recalled a close contact with a positive COVID19 case before having a new positive SARS-CoV-2 RNA detection assay. Interestingly, viral shedding of the newly acquired SARS-CoV-2 infection in patient 17 and 27 was very short compared with data from the first episodes, suggesting that an early reinfection may have low infectivity potential. Besides, participants with a probable reinfection did not have any symptoms, unlike what happened in the first episode when all of them have mild symptoms. Our data suggest that reinfection in highly exposed subjects with a mild first episode can appear within the 12 weeks after the first episode, supporting observation from other coronaviruses. Reinfections with seasonal coronaviruses have been reported even with the presence of high antibody titres [16]. In the HC cohort we did not find any newly acquired SARS-CoV-2 infection, suggesting that uninfected healthcare workers may act with higher precaution at hospital and in the community, limiting the chances of acquiring a SARS-CoV-2 infection.

In a population-based study conducted in Qatar, newly acquired SARS-CoV-2 infection in patients with previous history of infection in the last 6 months was 0.02% and the incidence reinfection rate was 0.36 per 10,000 person-week [17]. These results should be interpreted with caution, since asymptomatic patients were not systematically tested. A recent publication in health care workers found an incidence reinfection rate of 1.09 per 10,000 days at risk in anti-Spike seronegative participants and 0.13 per 10,000 person-day in anti-Spike seropositive participants [10]. Our study depicts a highly exposed population of health care workers dealing with the peak of the first wave of the pandemic in Spain [18]. Although, our results may not be informative of the risk of reinfection in the community, they highlight the possibility of early newly acquired infections even with the presence of antibodies. Many healthcare workers during the first pandemic wave acquired the infection in the community, although hospital acquired infections were more frequent in workers caring from COVID19 patients [19].

Outstanding efforts to develop a vaccine with long-term protection against SARS-CoV-2 infection have been made during the last months. Currently several vaccines against SARS-CoV-2 are being administered worldwide, however the long-term protective effects are still unknown [20]. The success of the vaccine relies on the long-term persistence of neutralizing antibodies and the development of immune memory cells [21]. It has been proposed that high titres of neutralizing antibodies could prevent infections or rapidly sterilize the virus. Furthermore, neutralisation activity has been associated with high anti-receptor-domain antibodies titres 3 weeks after the infection [22,23]. Unfortunately, first reports and experiences from previous coronaviruses suggest waning over time [21,24]. To date, between 6 to 8 months after symptoms onset 90% of the infected patients had detectable spike-binding IgG antibodies. On the other hand, disease severity is expected to depend, to a large degree, on the persistency of immune memory cells [25].

From a public health point of view, knowing the potential infectivity of reinfected patients is of utmost importance to design intervention strategies. There is little information regarding SARS-CoV-2 transmission blocking effect in previously immunized subjects, however no cases of secondary infection have been described from sequence-proven reinfection cases. Our data suggest that SARS-CoV-2 RNA detection long after symptoms onset is possible, regardless the presence of IgG antibodies, although none of this reinfection led to symptoms or secondary cases, opening a window of hope that vaccination could wane the impact of the pandemic.

Several limitations of this study should be mentioned. Firstly, the time of the sample collection between the first episode and the first follow-up nasopharyngeal swab were slightly heterogeneous among participants. However, all of them except one were recruited within the first month after the first PCR-proven episode. Secondly, the sample size of the study was calculated assuming a higher infection rate among uninfected professionals. Surprisingly, our results did not show any infection in the HC group. Accordingly, our study was not aimed to study the incidence rate of reinfection. Another limitation was the insufficient sample volume or the low viral load to perform sequencing studies of the patients suspected to be re-infected, so absolute certainty of reinfection cannot be obtained. However, the time lapse between the first infection and the posterior positive molecular test eliciting a probable newly acquired infection, the evidence of negative results in between, and CT results from PCR tests support our interpretation of newly acquired infections. Persistent infection in our cases is unlikely due to the resolution of symptoms and negative results between episodes, limiting the possibility of viral remnants. We did not assess infectivity by means of viral viability in cell culture isolation, which prevent us from knowing whether reinfections have the potential to infect other subjects. It is noteworthy that our participants were young healthcare workers, and the subjects from IC group had mild symptoms during the acute initial episode, other populations may behave differently. In support of our study is the used of TMA tests, which have shown greater sensitivity than the conventional RT-PCR [26]. Subsequent cohort studies with healthcare workers should consider sequencing and infectivity studies, as well as prolonged follow up schemes with regular respiratory and blood samples.

## 5. Conclusions

Our study shows SARS-CoV-2 serology and RNA detection dynamic in previously infected and uninfected cohorts of healthcare workers caring for COVID19 patients. Despite, detectable IgG antibodies against SARS-CoV-2, participants highly exposed to the virus may develop newly acquired SARS-CoV-2 RNA detection episode during the first months after the initial infection. These results may help to design vaccination strategy and highlight the need of a follow up of the pandemic transmission dynamic.

## Figures and Tables

**Table 1:** Demographic characteristics of the cohort.

	HC (*n* = 20)	IC Cohort (*n* = 20)
Age, years	29 (26–36.75)	30 (28–34)
Sex, female	12 (60%)	10 (50%)
Immunosuppression	0	1 (5%)
Caring for COVID19 patients	100%	100%
Emergency department shifts	100%	100%
Role in the hospital		
Physician	18 (90%)	19 (95%)
Nurse	2 (10%)	1 (1%)
Time from first molecular test to inclusion, days	-	22.5 (18–25)
Follow up time, days	49 (49–51)	49 (49–49)

Acronyms: HC, healthy cohort; IC, infected cohort.

## Data Availability

Data can be obtained upon request to the corresponding author.

## Appendix A

Detailed information of the participant with suspected SARS-CoV-2 reinfection.

### Case Description of the Probable Reinfection Cases

Participant 4 had a positive SARS-CoV-2 PCR from a nasopharyngeal swab on March 20th. His symptoms started 2 days before the swab collection and consisted in high fever, asthenia, headache, myalgia, diarrhoea and anosmia. All symptoms resolved in 10 days. The participant did not recall contact with COVID19 patients outside the Hospital. The participant had 3 consecutive positive SARS-CoV-2 RNA detection assays after symptoms resolved. Last positive SARS-CoV-2 RNA detection assay was on April 29th. Afterward, the samples on May 5th and May 13th were negative. The last SARS-CoV-2 RNA detection assay on May 25th was again positive. The participant did not report any symptom or secondary cases.

Participant 5 started had viral symptoms on March 21st including asthenia, headache, arthromyalgia, low-grade fever and anosmia. Symptoms resolved in 4 days, except for anosmia that lasted for up to 4 months. This participant showed a possible episode of reinfection on May 23th due to a SARS-CoV-2 RNA detection assay by TMA. This sample could not be retested by RT-PCR due to insufficient volume. The participant did not report any symptom or secondary cases derived from the last positive result.

The participant 8 showed anosmia on March 26th that was resolved after 7 days but reported having thoracic ache for one month. During the next 2 months the participant had 8 weekly negative SARS-CoV-2 RNA detection assays. Five months later, this participant reported having had close contact with a person infected with SARS-CoV-2 and was retested with TMA test that was positive. The participant had detectable IgG antibodies at least from June 6th, and were retested during the reinfection episode and persisted positive. The participant was not aware of secondary cases, and reported no symptoms.

The patient 17 started viral symptoms on March 22nd and lasted for 10 days. Two months after the diagnosis and after five negative samples during the follow-up (confirmed by RT-PCR), he had two consecutive SARS-CoV-2 RNA detection assay (one by both TMA and RT-PCR and one by TMA). Again, no symptoms or secondary cases were reported. IgG antibodies at the beginning of the inclusion were positive, the second determination two months later showed indeterminate results.

The participant 27 had low-fever and anosmia on March 31st. Anosmia lasted for two weeks. During the next 2 months the participant had 8 weekly negative SARS-CoV-2 RNA detection assays. IgG antibodies against SARS-CoV-2 were detected neither at the initial nor at the end of the follow up. Six months later, during a hospital massive screening the participant had a positive for SARS-CoV-2 infection by TMA. The participant had no other sign of infection. There were no secondary cases from the second episode. One month later, the IgG antibodies remained negative.

Participant 33 had symptoms on March 11th consisting in high fever, myalgias and cough. Symptoms resolved in one week. On March 24th participant was included in the study and had a negative RNA SARS-CoV-2 detection assay and positive IgG antibodies against SARS-CoV-2. On May 8th the participant had a positive RNA SARS-CoV-2 detection assay by TMA. Again no symptoms or secondary cases were reported. At the end the 7-week period the participant persisted with positive IgG antibodies against SARS-CoV-2.
